# Research on the Thermal Insulation Properties of Three-Dimensional Spacer Jacquard Fabric Treated with Silica Aerogel

**DOI:** 10.3390/ma16216954

**Published:** 2023-10-30

**Authors:** Zhifang Zhou, Jiu Zhou, Shuangyi Lu

**Affiliations:** Key Laboratory for Advanced Textile Materials and Manufacturing Technology, Ministry of Education, Zhejiang Sci-Tech University, Hangzhou 310018, China; 15068105756@163.com (Z.Z.);

**Keywords:** 3D spacer Jacquard fabric, silica aerogel, thermal insulation

## Abstract

Three-dimensional (3D) spacer fabric has the characteristics of a light weight and high strength, and its unique three-dimensional structure gives it great potential for development in terms of insulation. For the purpose of further improving the thermal insulation performance of 3D spacer fabric, the fabric was treated with silica aerogel while solving the problem of powdering during use. Firstly, the electronic Jacquard machine was modified for weaving spacer fabrics. The ground warp yarns were controlled by two groups of heald frames with various positions of heald eye, forming a double-shuttle. The longitudinal warp yarns were controlled by the Jacquard healdwine to prepare a spacer Jacquard fabric with a spacing of 5 mm. Secondly, polyurethane foam was applied as a carrier to compound with silica aerogel. The experimental results demonstrate that the strength of the composite fabrics is significantly increased, while the toughness is decreased. With the increase in silica aerogel content, the pore size of foam becomes smaller, and the degree of foam fragmentation increases, showing a trend of increasing thermal insulation performance followed by a decreasing insulation performance. When the aerogel content is 3.3%, the composite fabric has the optimal thermal insulation performance.

## 1. Introduction

With the growing issue of energy shortage, energy conservation and emission reduction are becoming increasingly important. The use of thermal insulation materials is one of the main measures for energy conservation and emission reduction [1]. Thermal insulation material, commonly referred to as composite materials, have a blocking effect on heat flow and achieve the purpose of thermal insulation, or cold insulation by impeding the heat exchange between objects and the outside [2]. With the development of the industry and the improvement in people’s living standards, such a kind of single thermal insulation material can no longer meet the market demand. In addition to thermal insulation, materials are also required to have characteristics such as being flame retardant, high strength, and light weight. The preparation of multi-functional composite thermal insulation materials is of great significance to meet the market demand [3,4].

Three-dimensional spacer fabrics are formed by three-dimensional weaving machines, with a good structural design, overall structure, and an anti-layering ability [5]. The weaving methods of 3D spacer fabric mainly include weaving and knitting. Three-dimensional woven fabric has the characteristics of a low weaving cost, good structural design, good resin permeability, and impact resistance [6,7]. Through reasonable structural design, 3D spacer fabric composites can be lightweight, featuring high strength, high modulus, thermal insulation, pressure resistance, and impact resistance, with a relatively large void ratio and good application prospects in the field of thermal insulation [8,9]. Current research mainly focuses on compounding other materials with 3D spacer fabric through coating [10], impregnation [11], and filling [12]. However, composite materials made by coating and impregnation are prone to aging and cracking during use, while composite fabrics made by filling often have problems with uneven distribution [13,14].

Silica aerogel is solid, but more than 96% of the composition is gas, so the density can be as low as 3 mg/cm^3^, the porosity can reach 80–99.8%, and the lowest thermal conductivity at room temperature is 0 013 W/(m·K) [15], with broad application prospects in the fields of heat preservation, sound insulation, adsorption, and so on [16,17]. Nonetheless, silica aerogel is brittle and has poor mechanical properties, which limit its large-scale promotion and application [18]. Currently, a great deal of research is conducted to improve the mechanical properties of aerogels by compounding aerogels with other materials: the main products are silica aerogel felts [19] and silica aerogel coatings [20]. The aerogel felt has a slag falling phenomenon in use. In addition, the aerogels powder for external use can accordingly easily fall off from the seam. With the addition of other organic substances, the function of aerogel itself will be limited and its thermal insulation performance will be reduced, and the durability of the coating is synchronously poor [21,22].

In this paper, the improved electronic Jacquard loom was used to weave 3D spacer Jacquard fabric, and the silica aerogel powder was mixed with polyurethane solution and filled into the 3D spacer Jacquard fabric for foaming. Polyurethane foam has the characteristics of a porous structure, low density, good adhesion, chemical resistance, and aging resistance [23]. This method can avoid the “powder running” problem of silica aerogel. Moreover, taking the 3D spacer Jacquard fabric as the skeleton, the mechanical properties of the composite material are effectively improved, and the silica aerogel composite 3D spacer Jacquard fabric with high strength, light weight, no powder loss, durability, and excellent thermal insulation performance is prepared. It is expected to be used as thermal insulation materials for buildings and automobiles.

## 2. Experimental

### 2.1. Preparation of 3D Spacer Jacquard Fabric

**Figure 1:**
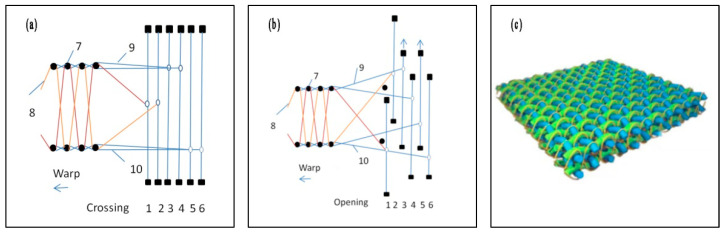
Schematic diagram of the interweaving of longitudinal warp and weft of 3D spacer Jacquard fabric: (**a**) shed closing; (**b**) shed opening; and (**c**) fabric structure diagram. 1,2—Jacquard healdwine; 3,4—heald frames; 5,6—heald frames; 7—weft yarns; 8—longitudinal warp yarns; 9—upper ground warp yarns; 10—lower ground warp yarns.

The 3D spacer fabric consists of three groups of yarns, the ground warp yarn forming the length direction of the fabric, the surface weft yarn forming the width direction of the fabric, and the longitudinal warp yarn forming the thickness direction of the fabric [24]. In this paper, the traditional SMIT electronic Jacquard machine was reformed with double shuttles. The ground warp yarns were controlled by two groups of heald frames with different heald eye positions, and the longitudinal warp yarns were controlled by the Jacquard healdwine. The longitudinal warp yarns were located between the upper and lower ground warp yarns, as illustrated in Figure 1a. Three groups of warp yarns were in different horizontal positions. The upper and lower parts were controlled by the heald frame, and the loom spindle rotates formed double sheds. As displayed in Figure 1b, 3 and 4 formed the upper shed, 5 and 6 formed the lower shed, and 1 and 2 were Jacquard threads to control the interweaving of longitudinal warp and ground weft yarns. By adjusting the opening height of the heald rope through the knife arm, when the loom was opened, the upper opening angle of the heald twine was flush with the upper part of the upper ground warp opening, and the lower opening angle was flush with the lower part of the lower ground warp opening, ensuring that the longitudinal warp yarns and the surface weft yarns were closely intertwined, and the weaving of the double-layer interval fabric was completed [25].

In production, the longitudinal warp yarns were placed on the bobbin frame, the longitudinal warp yarns were placed on the bobbin frame, and the ground warp with separate openings was driven by the warp beam. Longitudinal and ground warp yarns move at different speeds, so there were more contact points between warp yarns and machines, which were subject to greater friction. Ground warp yarns adopted active transmission, while longitudinal warp yarns adopted a negative let-off, which contribute to a reduction upon the friction of the warp yarns and facilitate weaving. The schematic diagram of the fabric interweaving structure is sketched in Figure 1c.

**Figure 2:**
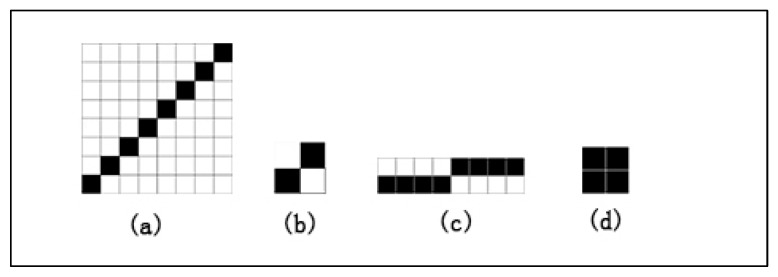
Looming drafts of 3D spacer Jacquard fabric: (**a**) drafting of ground warp; (**b**) chain draft; (**c**) reeding; and (**d**) weft selection.

The beam of ground warp obtains tension and uniform winding density through warping. The drawing-in process of ground warps could be separated. The first four pages of the harness controlled the upper ground warp opening, while the last four pages of harness controlled the lower ground warp opening. Heald twine Jacquard was located at the front end of the heald frame, where the longitudinal warp yarns needs to pass through the gap between the heddles. The weaving preparation of the 3D spacer Jacquard fabric was similar to 2D woven fabric. The main process included warping, drafting, reeding, weft preparation, the input of machine parameters, and weaving. The ground warp yarns were separately transmitted by two weaving shafts, with the number of ground warp and longitudinal warp count of 1:1. Looming drafts are shown in Figure 2. It adopted intermittent curling method and controlled interval distance of the spacer fabric by adjusting the warp feed amount. In this paper, a type of 3D fabric with a spacing of 5 mm was prepared by employing polyester as a raw material, and the weaving parameters ae shown in Table 1.

### 2.2. Preparation of Silica Aerogel Composite Spacer Jacquard Fabric

**Figure 3:**
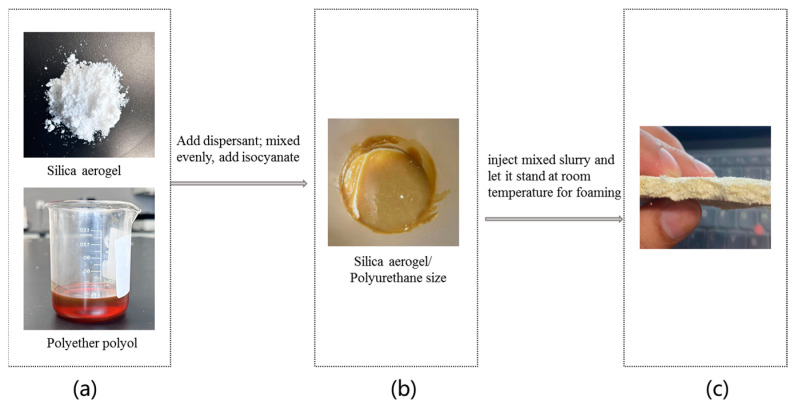
Diagram of preparation of silica aerogel/polyurethane foam composite 3D spacer Jacquard fabric: (**a**) Silica aerogel powder was added into the weighed polyether polyol; (**b**) Mixed size prepared by adding isocyanate; (**c**) Filling aerogel mixed slurry into the prepared 3D spacer Jacquard fabric with syringe.

Silica aerogel is produced by Kaihua Energy Conservation Technology Co., Ltd., Shijiazhuang, Hebei, China, and polyurethane is provided by Guangsheng Building Materials Co., Ltd., Shanghai, China. Firstly, the silica aerogel powder was added into the weighed polyether polyol, and subsequently an appropriate amount of dispersant was added, which was stirred evenly with a magnetic stirrer, and then ultrasonic dispersion treatment was carried out for 10 min. After mixing evenly, a quantitative isocyanate was added and stirred quickly for 30S. The process of filling the aerogel mixed slurry into the prepared 3D spacer Jacquard fabric with a syringe is shown in Figure 3. After the polyurethane foaming was completed at room temperature, the foam on the fabric surface was removed, and the 3D space Jacquard fabric with silica aerogel/polyurethane foam composite was made.

## 3. Testing Process

### 3.1. Mechanical Tests

This study uses a multifunctional electronic strength machine to test the tensile breaking strength of composite fabrics.

### 3.2. Characterizations

The surface and cross-section of the composite material were observed using optical microscope (Ningbo Sunny Instrum ents Co., Ltd., Ningbo, China). The microstructure of the samples was imaged by scanning electron microscope (Zeiss Sigma 300, Oberkochen, Germany) at a voltage of 10 KV. Before measuring, a layer of gold was coated on the fabric to produce electrical conductivity.

### 3.3. Thermal Insulation Tests

The thermal insulation properties of the 3D spacer Jacquard fabrics with different silica aerogel contents were tested by infrared thermography (Shanghai Hongce Intelligent Technology Co., Ltd., Shanghai, China). The bottom surface of the fabric was heated by a constant temperature heat source, and the temperature of the upper surface was captured by an infrared camera, from which the insulation efficiency of the sample could be calculated.

## 4. Results and Discussion

### 4.1. Mechanical Properties of Spacer Jacquard Fabric

**Figure 4:**
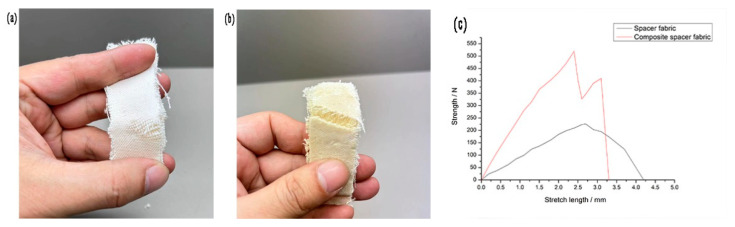
Mechanical properties of 3D spacer fabric and composite fabric: (**a**) Tensile fracture image of 3D spacer Jacquard fabric; (**b**) Tensile fracture image of 3D spacer Jacquard fabric with 3.3% silica aerogel; and (**c**) Tensile fracture curve of 3D spacer Jacquard fabric and composites fabric.

The blank spacer fabric and the composite fabric with 3.3% silica aerogel content are into 3 cm × 10 cm, and the clamping distance is 5 cm. It can be seen from the tensile fracture diagram that the spacer fabric is broken by the ground weave yarn, and the cracks are uneven, as shown in Figure 4a. The fracture of the composite fabric is neat, similar to the incision of the board, and the longitudinal warp yarn is not broken, as presented in Figure 4b. As can be seen from Table 2, the breaking strength of the composite fabric has increased by more than two times, and the breaking elongation of the composite fabric has decreased by approximately 29%. The weft breaking strength of the fabric is greater than the warp breaking strength. The weft tensile breaking curve of the fabric shows that the initial modulus of the composite fabric is also significantly higher than that of the 3D spacer fabric. The yarn on the surface of the composite fabric breaks first, and then the polyurethane foam in the interlayer breaks under tension, so there are two peaks in Figure 4c.

### 4.2. Morphological Analysis of Spacer Jacquard Fabric

#### 4.2.1. Optical Microscope Analysis

**Figure 5:**
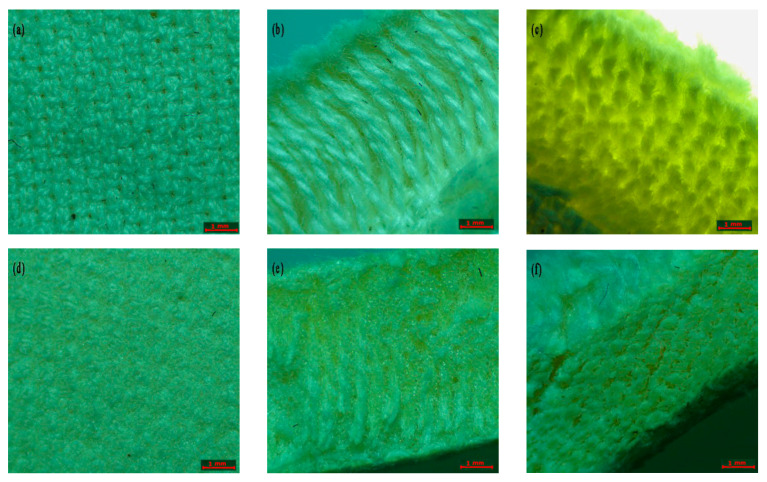
Optical microscope image of fabric: (**a**) surface of original spacer fabric; (**b**) longitudinal section of original spacer fabric; (**c**) cross section of original spacer fabric; (**d**) surface of composite spacer fabric; (**e**) longitudinal section of composite spacer fabric; and (**f**) cross-section composite spacer fabric.

The surface layer and cross-section of the composite fabric are sampled and analyzed using an optical microscope. As shown in Figure 5a, there are numerous regular pores formed by interweaving yarns on the surface of the 3D spaced Jacquard fabric. After silica aerogel/polyurethane composite, the surface pores are filled (Figure 5d). The air flow in the spacer fabric is reduced, thus improving the thermal insulation performance of the composite fabric. It can be seen from the cross-section of the composite fabric that the longitudinal warp of the spacer Jacquard fabric is wrapped by silica aerogel/polyurethane slurry (Figure 5e). After foaming, a closed hole is formed in the spacer layer to lock the aerogel powder, so as to avoid the powder loss of the composite fabric.

#### 4.2.2. SEM Analysis

**Figure 6:**
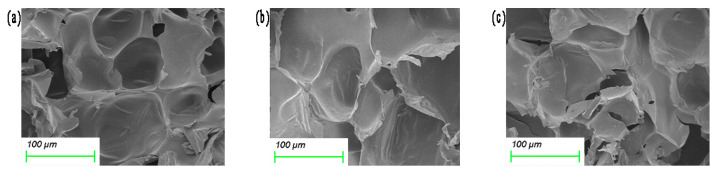

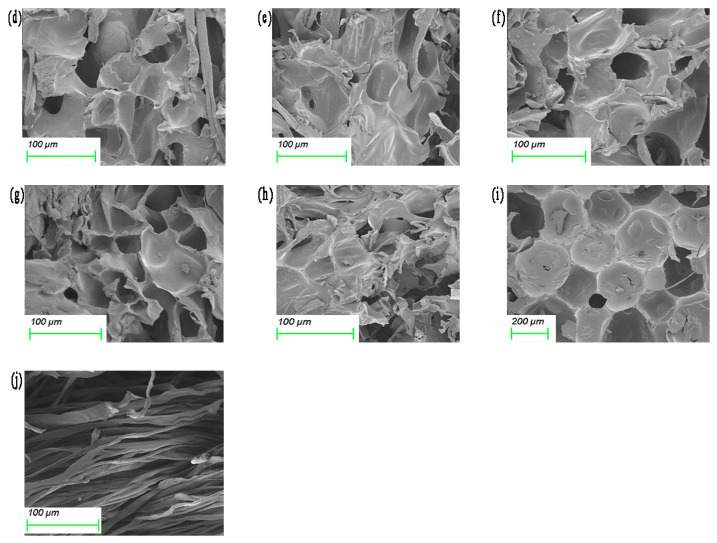
SEM of composite fabrics with different silica aerogel contents: (**a**) without aerogel; (**b**) 1.3% silica aerogel; (**c**) 3.3% silica aerogel; (**d**) 5.3% silica aerogel; (**e**) 6.7% silica aerogel; (**f**) 8% silica aerogel; (**g**) 10% silica aerogel; (**h**) 13.3% silica aerogel; (**i**) polyurethane foam of the same thickness; and (**j**) unfilled 3D spacer Jacquard fabric.

The SEM image of the sample is demonstrated in Figure 6. Figure 6i is a cross-sectional view of the polyurethane foam. It can be seen that the pore size of foam is evenly distributed, with a diameter of about 200 μm. From the electron microscope image of the composite spacer fabric, it can be seen that the pore size of the core layer of the spacer fabric is different, with pore diameters ranging from 30 to 100 μm. Figure 6j is an electron microscopic view of the 3D spacer Jacquard fabric. It can be seen that there is a large gap between the yarns. Figure 6a displays a polyurethane foam composite spacer Jacquard fabric without the silica aerogel, with a relatively uniform pore diameter of 80 μm and a foam hole wall completion. The pore diameter of foam decreases with the increase in aerogel dioxide content, mainly due to the fact that the increase in aerogel content leads to the increase in system viscosity, which makes the pore diameter of foam smaller. Moreover, the agglomeration will occur with the increase in aerogel content, and an uneven dispersion in the slurry will affect the nucleation of foam, as shown in Figure 6h. When the content of aerogel is 13.3%, the foam in the core layer is severely broken, which means fewer complete foam pore walls.

### 4.3. Thermal Insulation Performance

**Figure 7:**
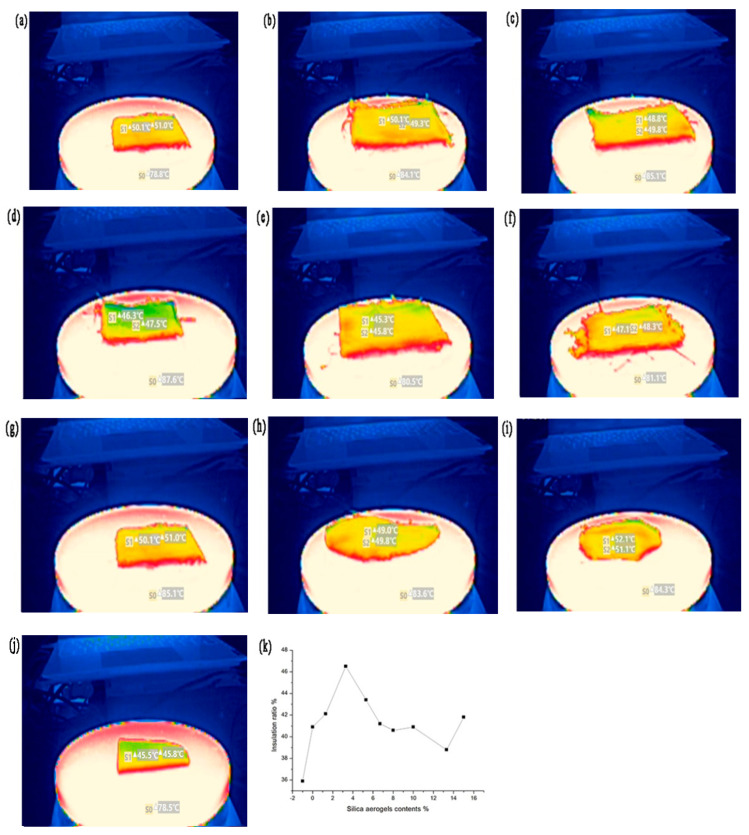
Thermal insulation properties of composite fabrics with different aerogel contents: (**a**) unfilled 3D spacer fabric; (**b**) without aerogel; (**c**) 1.3% silica aerogel; (**d**) 3.3% silica aerogel; (**e**) 5.3% silica aerogel; (**f**) 6.7% silica aerogel; (**g**) 8% silica aerogel; (**h**) 10% silica aerogel; (**i**) 13.3% silica aerogel; (**j**) polyurethane foam of the same thickness; and (**k**) thermal insulation graph of samples.

The fabric is placed on an electric heating platform, and the thermal insulation performance of the composite spacer fabrics with different silica aerogel content is tested by infrared camera. After heating to 80 °C, the surface temperature tends to be stable after 3 min. Then, the temperature difference is measured between the upper and lower surfaces of the sample. It can be seen that the thermal insulation performance of the sample is higher than 35%, as shown in Table 3. The thermal insulation performance of the 3D spacer Jacquard fabric is lower than 36%. As shown in Figure 7a, with the increase in silica aerogel content, the thermal insulation performance of the fabric first increases and decreases afterwards, reaching a peak of 46.5% when the silica aerogel content is 3.3%. As shown in Figure 7i, when the content is 13.3%, the insulation performance of the composite spacer fabric is close to that of the original spacer fabric. With the increase in silica aerogel content, it is easy to agglomerate and disperse unevenly in the mixed slurry, which affects the foaming of the system and reduces the thermal insulation performance of the composite fabric. The thermal insulation of the polyurethane foam board with the same thickness is 41.8%, which is higher than that of unfilled 3D spacer fabric, and lower than that of composite spacer fabric containing 3.3% silica aerogel.

## 5. Conclusions

This article introduces the thermal insulation performance of silica aerogel composite 3D spacer Jacquard fabric. The results show that silica aerogel can improve the thermal insulation performance of the composite fabric and increase the mechanical performance of the fabric. The specific results are summarized as follows:

(1) Through the modification of the Jacquard loom, the 3D spacer Jacquard fabric was successfully prepared. The silica aerogel was compounded with the spacer fabric using polyurethane foam as an intermediate. The breaking strength of the composite fabric increased from 200 CN to 519.3 CN, and the breaking elongation decreased by about 20%. The results of the optical microscope present that the structure of the composite fabric is compact, which avoids the powder dropping phenomenon of the silica aerogel during use. According to the SEM results, as the aerogel content increased, the system viscosity increased, leading to a reduction in the foam pore size. Furthermore, an increase in the aerogel content resulted in an agglomeration, uneven dispersion in the slurry, affecting foam nucleation. When the content of silica aerogel is 13%, the foam will be broken seriously.
(2) The insulation performance test results sketch that the insulation rate of the unfilled 3D spacing fabric is 35.9%. Only the thermal insulation rate of the polyurethane foam composite fabric is 40.9%, which is slightly lower than 41.8% of the polyurethane foam of the same thickness. When the content of silica aerogel is 3.3%, the thermal insulation effect is optimal, and the thermal insulation rate is 46.5%. With the increase in aerogel content, the thermal insulation performance of the composite fabric decreases. When the content of the silica aerogel is 13.3%, the thermal insulation rate is 38.8%, which is lower than the composite fabric without silica aerogel.

## Figures and Tables

**Table 1:** Weaving parameters.

Weaving Parameters	Ground Weave Warp	Ground Weave Weft	longitudinal Warp	Ground Weave Warp Density	Ground Weave Weft Density	Spacer Distances
Spacer fabric	28 tex polyester	28 tex polyester	28 tex polyester	22/cm	24/cm	5 mm

**Table 2:** Fabric strength test results.

Fabrics	Breaking Strength/N		Breaking Elongation/%	
	Warp Direction	Weft Direction	Warp Direction	Weft Direction
Spacer fabric	160.34	226.16	35.62	36.18
Spacer fabric with 3.3% silica aerogel	290.37	519.30	6.34	6.69

**Table 3:** Thermal insulation test results (content of −1 refers to unfilled spacer fabric); a content of 15 refers to the polyurethane foam of the same thickness).

No.	Silica Aerogel Content/%	Test Temperature/°C	Difference in Temperature/°C	Insulation Rate/%	CV%
a	−1 (blank)	78.8	28.3	35.9	0.9
b	0	84.1	34.4	40.9	0.8
c	1.3	85.1	35.8	42.1	1.0
d	3.3	87.6	40.7	46.5	1.3
e	5.3	80.5	35.0	43.4	0.5
f	6.7	81.1	33.4	41.2	1.3
g	8.0	85.1	34.6	40.6	0.9
h	10.0	83.6	34.2	40.9	0.8
i	13.3	84.3	32.7	38.8	1.0
j	15.0	78.5	32.9	41.8	0.3

## Data Availability

Not applicable.
